# Correction: ESCRT-I fuels lysosomal degradation to restrict TFEB/TFE3 signaling via the Rag-mTORC1 pathway

**DOI:** 10.26508/lsa.202201537

**Published:** 2022-06-07

**Authors:** Marta Wróbel, Jarosław Cendrowski, Ewelina Szymańska, Malwina Grębowicz-Maciukiewicz, Noga Budick-Harmelin, Matylda Macias, Aleksandra Szybińska, Michał Mazur, Krzysztof Kolmus, Krzysztof Goryca, Michalina Dąbrowska, Agnieszka Paziewska, Michał Mikula, Marta Miączyńska

**Affiliations:** 1 Laboratory of Cell Biology, International Institute of Molecular and Cell Biology, Warsaw, Poland; 2 Microscopy and Cytometry Facility, International Institute of Molecular and Cell Biology, Warsaw, Poland; 3 Department of Genetics, Maria Skłodowska-Curie National Research Institute of Oncology, Warsaw, Poland; 4 Department of Gastroenterology, Hepatology and Clinical Oncology, Medical Center for Postgraduate Education, Warsaw, Poland

Dear Editors

It came to our attention that in several places of the manuscript, the name of the S122 phosphorylation site in TFEB protein has been misspelled as S112. The errors and their proposed corrections are indicated below (corrections are needed in 13 places). We apologize for the inconvenience; this correction bears no impact on the meaning of the text.

Article: Wróbel M, Cendrowski J, Szymańska E, Grębowicz-Maciukiewicz M, Budick-Harmelin N, Macias M, Szybińska A, Mazur M, Kolmus K, Goryca K, Dąbrowska M, Paziewska A, Mikula M, Miączyńska M (2022 Mar 30) ESCRT-I fuels lysosomal degradation to restrict TFEB/TFE3 signaling via the Rag-mTORC1 pathway. *Life Sci Alliance* 5(7): e202101239. doi: 10.26508/lsa.202101239. PMID: 35354596; PMCID: PMC8967991.

In the Abstract

Where it reads:

However, we discovered that this activation occurs due to the inhibition of Rag GTPase–dependent mTORC1 pathway that specifically reduced phosphorylation of TFEB at **S112**. Constitutive activation of the Rag GTPase complex in cells lacking ESCRT-I restored **S112** phosphorylation and prevented TFEB/TFE3 activation.

It should read:

However, we discovered that this activation occurs due to the inhibition of Rag GTPase–dependent mTORC1 pathway that specifically reduced phosphorylation of TFEB at **S122**. Constitutive activation of the Rag GTPase complex in cells lacking ESCRT-I restored **S122** phosphorylation and prevented TFEB/TFE3 activation.

In the Results section

Where it reads:

ESCRT-I deficiency reduces TFEB phosphorylation at **S112** without having a broad effect on mTORC1 signaling

After excluding the involvement of endolysosomal cholesterol accumulation or Ca^2+^-dependent dephosphorylation, we investigated whether the induction of TFEB/TFE3 in ESCRT-I depleted cells occurs via regulation of mTORC1 kinase signaling. Having observed reduced phosphorylation of **S112** that is a direct mTORC1 target (Vega-Rubin-de-Celis et al, 2017), we investigated whether it occurs because of inhibition of general mTORC1 signaling, which would point to a broad starvation response in ESCRT-I–deficient cells (Ng et al, 2011). To this end, we compared the regulation of TFEB **S112** phosphorylation to that of other described mTORC1 kinase targets (Ulk1, S6K, or 4E-BP1), in control or ESCRT-I–depleted cells, under normal growth conditions (EMEM full medium) or upon nutrient deprivation (EBSS medium). In control cells, the phosphorylation signals of all tested targets were easily detected upon EMEM, and their levels were clearly reduced upon EBSS (Fig 7A and B), verifying that all of these phosphorylations are under constant activation in RKO cells. Importantly, the reduction of **S112** phosphorylation due to depletion of Tsg101 or Vps28 was as strong as observed for control cells upon EBSS (Fig 7A and B). However, ESCRT-I deficiency did not inhibit phosphorylations of other tested mTORC1 targets (Fig 7A and B). Reassuringly, we also observed reduced phosphorylation of TFEB at **S112** but not of Ulk1, S6K, or 4E-BP1 in cells lacking ESCRT-I because of CRISPR/Cas9–mediated Tsg101 depletion (Fig S7A; depletion efficiencies shown in Fig S1C). Consistent with no effect on the canonical mTORC1 targets, we found that ESCRT-I depletion did not alter the association of the mTOR protein with LAMP1-positive structures (Fig S7B) that is required for general mTORC1 signaling (Sancak et al, 2010), either upon normal growth conditions or when this association was reduced due to nutrient deprivation. Hence, we discovered a specific response to ESCRT-I deficiency that involves reduced **S112** phosphorylation of TFEB, independent of canonical mTORC1 signaling. This pointed to the inhibition of a recently identified mTORC1 pathway that specifically regulates MiT-TFE factors (Napolitano et al, 2020; Alesi et al, 2021).

It should read:

ESCRT-I deficiency reduces TFEB phosphorylation at **S122** without having a broad effect on mTORC1 signaling

After excluding the involvement of endolysosomal cholesterol accumulation or Ca^2+^-dependent dephosphorylation, we investigated whether the induction of TFEB/TFE3 in ESCRT-I depleted cells occurs via regulation of mTORC1 kinase signaling. Having observed reduced phosphorylation of **S122** that is a direct mTORC1 target (Vega-Rubin-de-Celis et al, 2017), we investigated whether it occurs because of inhibition of general mTORC1 signaling, which would point to a broad starvation response in ESCRT-I–deficient cells (Ng et al, 2011). To this end, we compared the regulation of TFEB **S122** phosphorylation to that of other described mTORC1 kinase targets (Ulk1, S6K, or 4E-BP1), in control or ESCRT-I–depleted cells, under normal growth conditions (EMEM full medium) or upon nutrient deprivation (EBSS medium). In control cells, the phosphorylation signals of all tested targets were easily detected upon EMEM, and their levels were clearly reduced upon EBSS (Fig 7A and B), verifying that all of these phosphorylations are under constant activation in RKO cells. Importantly, the reduction of **S122** phosphorylation due to depletion of Tsg101 or Vps28 was as strong as observed for control cells upon EBSS (Fig 7A and B). However, ESCRT-I deficiency did not inhibit phosphorylations of other tested mTORC1 targets (Fig 7A and B). Reassuringly, we also observed reduced phosphorylation of TFEB at **S122** but not of Ulk1, S6K, or 4E-BP1 in cells lacking ESCRT-I because of CRISPR/Cas9–mediated Tsg101 depletion (Fig S7A; depletion efficiencies shown in Fig S1C). Consistent with no effect on the canonical mTORC1 targets, we found that ESCRT-I depletion did not alter the association of the mTOR protein with LAMP1-positive structures (Fig S7B) that is required for general mTORC1 signaling (Sancak et al, 2010), either upon normal growth conditions or when this association was reduced due to nutrient deprivation. Hence, we discovered a specific response to ESCRT-I deficiency that involves reduced **S122** phosphorylation of TFEB, independent of canonical mTORC1 signaling. This pointed to the inhibition of a recently identified mTORC1 pathway that specifically regulates MiT-TFE factors (Napolitano et al, 2020; Alesi et al, 2021).

Where it reads:

Importantly, the inhibition of TFEB/TFE3 nuclear translocation by the active RagC mutant, in control and Tsg101-depleted cells, was associated with a strong induction of TFEB **S112** phosphorylation (Fig 7G), although levels of Ulk1 or S6K phosphorylations remained unaffected.

It should read:

Importantly, the inhibition of TFEB/TFE3 nuclear translocation by the active RagC mutant, in control and Tsg101-depleted cells, was associated with a strong induction of TFEB **S122** phosphorylation (Fig 7G), although levels of Ulk1 or S6K phosphorylations remained unaffected.

In the legend for Fig 7

Where it reads:

(A) Western blots showing levels of phosphorylation of TFEB at **Ser112** and the indicated canonical mTORC1 targets in cells lacking ESCRT-I (transfected with siTsg101#2 or siVps28#1) as compared with control cells (siCtrl#1 or #2, nontargeting siRNAs) at 48 h post transfection (48 hpt) upon culture in regular medium (EMEM) or nutrient-deficient medium (EBSS) for 2 h.

It should read:

(A) Western blots showing levels of phosphorylation of TFEB at **Ser122** and the indicated canonical mTORC1 targets in cells lacking ESCRT-I (transfected with siTsg101#2 or siVps28#1) as compared with control cells (siCtrl#1 or #2, nontargeting siRNAs) at 48 h post transfection (48 hpt) upon culture in regular medium (EMEM) or nutrient-deficient medium (EBSS) for 2 h.

Where it reads:

(G) Western blots showing levels of phosphorylation of the indicated proteins as well as total levels of TFEB, Tsg101, and HA-GST-RagC in ESCRT-I–depleted (siTsg101#2) or control (siCtrl#1) cells at 48 hpt with ectopic expression of the HA-GST-RagC protein (WT or S75L). Vinculin was used as a loading control. The bottom graph shows phosphorylation levels of **Ser112** of TFEB measured by densitometry analysis of Western blotting bands, including those shown above.

It should read:

(G) Western blots showing levels of phosphorylation of the indicated proteins as well as total levels of TFEB, Tsg101, and HA-GST-RagC in ESCRT-I–depleted (siTsg101#2) or control (siCtrl#1) cells at 48 hpt with ectopic expression of the HA-GST-RagC protein (WT or S75L). Vinculin was used as a loading control. The bottom graph shows phosphorylation levels of **Ser122** of TFEB measured by densitometry analysis of Western blotting bands, including those shown above.

In the legend for Fig S7

Where it reads:

ESCRT-I deficiency inhibits TFEB **S112** phosphorylation and activates TFEB/TFE3 factors independently of general mTORC1 signaling.

(A) Representative Western blots showing levels of phosphorylation of TFEB at **Ser112** and phosphorylations of the indicated canonical mTORC1 targets

It should read:

ESCRT-I deficiency inhibits TFEB **S122** phosphorylation and activates TFEB/TFE3 factors independently of general mTORC1 signaling.

(A) Representative Western blots showing levels of phosphorylation of TFEB at **Ser122** and phosphorylations of the indicated canonical mTORC1 targets

## Supplementary Material

Reviewer comments

