# Supplementary material for: Correction: ESCRT-I fuels lysosomal degradation to restrict TFEB/TFE3 signaling via the Rag-mTORC1 pathway
Source: Life Sci Alliance. 2022 Jun 7;5(10):e202201537. doi: 10.26508/lsa.202201537 (PMC9175455; doi:10.26508/lsa.202201537)
Supplement: Reviewer comments [file LSA-2022-01537_review_history.pdf]

Life Science Alliance

Correction: ESCRT-I fuels lysosomal degradation to restrict TFEB/TFE3 signaling via the Rag-mTO[...]

Marta Wróbel, Jarosław Cendrowski, Ewelina Szymańska, Malwina Grębowicz-Maciukiewicz, Noga Budick-Harmelin, Matylda Macias, Aleksandra Szybinska, Michał Mazur, Krzysztof Kolmus, Krzysztof Goryca, Michalina Dąbrowska, Agnieszka Paziewska, Michał Mikula, and Marta Miaczynska
DOI: https://doi.org/10.26508/lsa.202201537

Corresponding author(s): Marta Miaczynska, International Institute of Molecular and Cell Biology and Jarosław Cendrowski, International Institute of Molecular and Cell Biology

**Review Timeline:**	Submission Date:	2022-05-27
	Accepted:	2022-05-27

Scientific Editor: Eric Sawey, PhD

Transaction Report:

(Note: With the exception of the correction of typographical or spelling errors that could be a source of ambiguity, letters and reports are not edited. The original formatting of letters and referee reports may not be reflected in this compilation.)

May 27, 2022

RE: Life Science Alliance Manuscript #LSA-2022-01537

Prof. Marta Miaczynska
International Institute of Molecular and Cell Biology
Laboratory of Cell Biology
Ks. Trojdena 4
Warsaw 02-109
Poland

Dear Dr. Miaczynska,

Thank you for submitting your Correction entitled "Correction: ESCRT-I fuels lysosomal degradation to restrict TFEB/TFE3 signaling via the Rag-mTO[...]".

The final published version will be deposited by us to PubMed Central upon online publication.

Your manuscript will now progress through copyediting and proofing.

IMPORTANT: If you will be unreachable at any time, please provide us with the email address of an alternate author. Failure to respond to routine queries may lead to unavoidable delays in publication.

You can contact the journal office with any questions, contact@life-science-alliance.org

Sincerely,

Eric Sawey, PhD
Executive Editor
Life Science Alliance
http://www.lsajournal.org

May 27, 2022

RE: Life Science Alliance Manuscript #LSA-2022-01537

Prof. Marta Miaczynska
International Institute of Molecular and Cell Biology
Laboratory of Cell Biology
Ks. Trojdena 4
Warsaw 02-109
Poland

Dear Dr. Miaczynska,

Thank you for submitting your Correction entitled "Correction: ESCRT-I fuels lysosomal degradation to restrict TFEB/TFE3 signaling via the Rag-mTO[...]".

The final published version will be deposited by us to PubMed Central upon online publication.

Your manuscript will now progress through copyediting and proofing.

IMPORTANT: If you will be unreachable at any time, please provide us with the email address of an alternate author. Failure to respond to routine queries may lead to unavoidable delays in publication.

You can contact the journal office with any questions, contact@life-science-alliance.org

Sincerely,

Eric Sawey, PhD
Executive Editor
Life Science Alliance
http://www.lsajournal.org
